# Organizational Justice, Professional Identification, Empathy, and Meaningful Work During COVID-19 Pandemic: Are They Burnout Protectors in Physicians and Nurses?

**DOI:** 10.3389/fpsyg.2020.566139

**Published:** 2020-12-11

**Authors:** Isabel Correia, Andreia E. Almeida

**Affiliations:** ^1^Departamento de Psicologia Social e das Organizações, Instituto Universitário de Lisboa (ISCTE-IUL), CIS-IUL, Lisbon, Portugal; ^2^Instituto Universitário de Lisboa (ISCTE-IUL), CIS-IUL, Lisbon, Portugal

**Keywords:** burnout, organizational justice, professional identification, social identity, empathy, meaningful work, workload, health care workers

## Abstract

Burnout has been recognized as a serious health problem. In Portugal, before COVID-19 Pandemic, there were strong indicators of high prevalence of burnout in physicians and nurses. However, the Portuguese Health Care Service was able to efficiently respond to the increased demands. This study intends to understand how psychosocial variables might have been protective factors for burnout in physicians and nurses in Portugal. Specifically, we considered several psychosocial variables that have been found to be protective factors for burnout in previous research and we compared their predictive and unique impact in the prediction of burnout. These variables are perceptions of justice (distributive, procedural, justice from colleagues, justice from patients, and their families), professional identification, meaningful work and empathy. We also included workload, as a risk factor, and controlled other variables that can be confounds for burnout, such as socio-demographic variables, ideological variables (religiosity, political orientation), and specific variables related with COVID-19 pandemic. The sample of the present study is composed by 229 physicians (aged between 23 and 70 years old, *M* = 36.54; *SD* = 10.72; 48% male and 52% female) and 268 nurses (aged between 22 and 69 years old, *M* = 34.96; *SD* = 9.52; 27% male and 73% female). An online survey was created using Qualtrics and participants were recruited via Facebook and LinkedIn. The data were collected during 29 days (between the 45th and the 74th days after the first diagnosed case of COVID-19 in Portugal). The results showed that workload was a significant risk factor, except for disengagement in physicians. The most consistent protectors across samples were procedural justice (for both dimensions of burnout, both in physicians and nurses) and professional identification (for disengagement, both in physicians and nurses; for exhaustion only in physicians). This study suggests that decreasing workload and promoting procedural justice and professional identification are key factors that might be simultaneously and independently addressed in interventions for reducing the risk of burnout or preventing it from occurring in the first place.

## Introduction

Burnout has been defined as a psychological syndrome of exhaustion, cynicism, and inefficacy resulting from ongoing occupational stressors (Leiter and Maslach, 2003), and that can take place in any kind of occupation (Leiter and Schaufeli, 1996).

Burnout has been recognized as a serious health problem and, particularly, the high incidence of burnout in physicians (West et al., 2018, for a review) and nurses (Woo et al., 2020, for a review) has been recognized as a threat, not only to the professionals themselves but also to their patients and the organizations in which they work. Indeed, burnout has been found to be associated with decreased mental and physical health of health care workers; lower quality care, threats to patient safety, and lower patient satisfaction; and reduced productivity, increased turnover and increased costs of the health care system (for a review in physicians see West et al., 2018; for a review in nurses see Bakhamis et al., 2019).

In Portugal, before COVID-19 Pandemic, there were strong indicators of high prevalence of burnout in physicians and nurses. In a study with a national sample of 9,176 of Portuguese physicians, it was found that 66% of them were in a high level of emotional exhaustion (Vala et al., 2017). In a sample of 1,262 nurses, also at a national level, about 50% had a high level of burnout (Marôco et al., 2016).

However, as stated by the President of the Order of Physicians (Guimarães, 2020), the Portuguese Health Care Service (Serviço Nacional de Saúde—SNS), was able to efficiently respond to the increased demands of the COVID-19 Pandemic. This may be considered somehow surprising. Indeed, despite the high levels of burnout already present in health care workers (Vala et al., 2017), they had to deal with the increased stress caused by the Pandemic (Bavel et al., 2020), and specifically as health care workers they faced additional stressors, such as increased workload, high risks of contagion and, many of them, isolation from their own families (Guimarães, 2020).

Research has shown that perceptions of justice, social identification, meaningful work and empathy are associated with burnout. However, that same research have been conducted under separate theoretical frameworks and the impact of these different predictors have not been tested together. Moreover, those same studies have not consistently included other variables related with work, socio-demographic variables and ideological variables, that can also impact on burnout.

The present paper has two main goals. The first goal is to identify the core psychosocial variables might have been protective factors for burnout in physicians and nurses in the first 2 months of the COVID-19 pandemic in Portugal, comparing their predictive and unique impact in the prediction of burnout. The variables considered were perceptions of justice (distributive, procedural, justice from colleagues, justice from patients, and their families), professional identification, meaningful work, and empathy. We also controlled for workload as a risk factor, and for other variables that can be confounds for burnout, such as socio-demographic variables (age, sex, income), ideological variables (religiosity, political orientation), and specific variables related with COVID-19 pandemic. The second goal is to understand how these variables might relate theoretically to explain burnout.

In the next section we will briefly review the literature related with each of the theoretical variables considered.

### Burnout

Burnout was first described by Freudenberger (1974) and mostly developed by Maslach (1976) in collaboration with other researchers (Schaufeli et al., 2009). It has been conceived as a cumulative reaction to ongoing occupational stressors and defined originally as a three-dimensional psychological syndrome of exhaustion, cynicism, and inefficacy (Leiter and Maslach, 2003): the exhaustion component refers to feelings of being overextended and depleted of one’s emotional and physical resources; cynicism component (also known as depersonalization or disengagement) refers to a negative, callous, or excessively detached response to various aspects of the job, that is self-protective of exhaustion, and can result in the loss of idealism and the dehumanization of others; the inefficacy refers to feelings of incompetence and a lack of achievement and productivity at work.

Later on, a two-dimensional approach emerged with exhaustion and cynicism as the two core dimensions of burnout, with inefficacy being considered as a possible consequence of burnout (e.g., Bakker et al., 2004). This is the approach we will be using in the present study (Oldenburg Burnout Inventory, OLBI, Bakker et al., 2004), with the two dimensions of burnout named as exhaustion and disengagement. The exhaustion dimension refers to feelings of physical fatigue and overload in relation to work (Demerouti and Bakker, 2008). The dimension of disengagement refers to the distance from work and negative attitudes toward own work (Bakker et al., 2004).

### Workload

The workload refers to overload, when job demands exceed human limits (Maslach et al., 2001). It is one of the core risk factors for burnout development when it is a chronic job condition and not an occasional emergency (Leiter and Maslach, 2003).

Both the Six Areas of Work Life Model (Leiter and Maslach, 2003; Brom et al., 2015) and the Job Demands—Resources Theory (Demerouti et al., 2001), consider workload a key variable to explain burnout.

### Justice Perceptions

Since the genesis of the Social Justice theories (Stouffer et al., 1949; Adams, 1965), perceived justice has been found to be an important predictor of satisfaction and well-being. More recently, organizational justice has been identified as an important predictor of health (Elovainio et al., 2002b) and of burnout (Maslach et al., 2001). Several dimensions of justice have been considered: distributive, procedural and interactional.

Distributive justice refers to the perception that the resources that are allocated to people are “deserved” or not, according to their contributions (Adams, 1965). If the reward obtained is proportional to the contribution, the situation is considered as just; if not, it is considered as unjust. The judgment of fairness may be comparative, with another person or with the same person in the past, or may be done in absolute terms.

Procedural justice refers to the fairness of the means by which distributions, or decisions about them, are made (Thibaut and Walker, 1975). Apart from the possibility of “having a voice” (Thibaut and Walker, 1975) in this process, a fair procedure has to be based on accurate information; and the patterns and criteria for decision-making have to be consistent (across people and time) and there should be a possibility of reversing decisions (Leventhal, 1980).

Interactional justice (Bies and Moag, 1986) refers to the respectful and proper manner by which authorities communicate procedural details and justify their decisions using honest and truthful information.

Not much studies have assessed and compared the unique impact of these three dimensions of organizational justice in burnout. With the intention of contributing to fill this gap, Moliner et al. (2005) found that distributive, procedural and interactional justice where all negatively associated with both cynicism and exhaustion but when considered together only procedural justice was a significant predictor of both dimensions of burnout. We believe that one reason for this might have been the high correlation between procedural and interactional dimensions. In this case, it is recommended to aggregate the measure in one procedural/interactional dimension, so that it is possible to avoid the costs of multicollinearity (Colquitt, 2012). This aggregation of the procedural and interactional justice in only one dimension is also in agreement with the Group Value Model (Tyler and Lind, 1992; Tyler, 1994) that includes both procedural and interactional aspects of justice in the conceptualization of a unique dimension named simply “procedural justice.”

The impact of justice perceptions on well-being can also be conceptualized as a buffer (Bobocel and Hafer, 2007) that serves to protect individuals of major stressors and decreases the impact of demands (a moderator hypothesis between workload and justice concerns).

In the present study we included other dimensions of justice that we think are particularly relevant for physicians and nurses, such as justice of colleagues, patient justice and family patient’s justice. Previous studies in physicians (Smets et al., 2004) showed that perceived injustice from colleagues was associated with exhaustion, and perceived injustice from patients was associated with both exhaustion and depersonalization. As far as we know, no previous studies have addressed the impact of perceived patient family justice on burnout. However, it was found that aggressive behavior (incivilities) from patients and their families, which can be considered as a proxy of perceived injustice, was associated with burnout (Campana and Hammoud, 2013).

### Professional Identification

Since the first studies that experimentally showed the impact of intergroup categorization (Tajfel et al., 1971), the concept of social identity and its implications for intergroup relations and well-being started to get attention from researchers.

Tajfel (1978) defined social identity as “that part of an individual’s self-concept which derives from his [or her] knowledge of his [or her] membership of a social group (or groups) together with the value and emotional significance attached to that membership” (p. 63). People belong to a variety of groups, and they may differ in the strength of the sense of membership, which is conceptualized as social identification (Turner et al., 1987). This strong sense of membership with a social group (that at the organizational level may be either the organization or the work team), has shown to be an important protector of health and well-being (Jetten et al., 2012) and an important protector for burnout (Avanzi et al., 2015, 2018).

The relation between social identification and burnout has been conceptualized in two different ways: social identity mediating the relation between procedural justice and well-being (self-esteem, the group-value model, Tyler et al., 1996) and social identification predicting a reduction in workload which is turn reduces burnout (Avanzi et al., 2018).

Because, in Portugal it is very common that physicians and nurses work in more than one organization, in this study, instead of identification with organization, we considered a measure of identification with the profession.

### Meaningful Work

Hackman and Oldham (1976) defined meaningfulness of the work as “the degree to which the employee experiences the job as one which is generally meaningful, valuable, and worthwhile” (Hackman and Oldham, 1976, p. 256). Although more recent conceptualizations of meaningful work have been proposed (Pratt and Ashforth, 2003; Steger et al., 2012) they incorporate the essence of this definition.

Meaningful work has mostly found to be an important protector of well-being at work (Duffy et al., 2012; Steger et al., 2012; Yaseen, 2013) and burnout (e.g., Borritz et al., 2005; Fouché et al., 2017), including burnout in physicians (Rasmussen et al., 2015) and nurses (Tei et al., 2014). However, recently Jones and Griep (2018) showed that meaningful work can also be a risk factor for burnout because it may lead employees to continue increasing their efforts beyond their limits.

### Empathy

Empathy is generally considered to be a two-dimensional construct with an affective dimension and a cognitive dimension (Mehrabian, 1997). The affective dimension refers to the capability to share another person’s emotional state (Eisenberg and Strayer, 1987), and the cognitive dimension of empathy, refers to the ability to understand (not necessarily share) another’s emotional state (Davis, 1994).

Both emotional and cognitive empathy of physicians and nurses have been found to be beneficial for the quality of care and for patient satisfaction (Wilkinson et al., 2017; Samra, 2018, for reviews). However, there is less agreement about the benefits of empathy for health care workers, with two competing hypothesis, that empathy might be either a protective or a risk factor for burnout (Zenasni et al., 2012). The results are indeed not conclusive. In a recent systematic review of 10 studies correlating empathy and burnout in health care professionals (Wilkinson et al., 2017), eight of the studies provided empirical support for a negative relationship between empathy and burnout, one study provided support for a positive relationship between burnout and empathy, and one study reported contradictory evidence with positive and negative correlations between different subscales of the empathy and burnout measures.

In this study we will try to contribute to address this issue and we will consider affective and cognitive empathy as distinct dimensions of empathy.

### Control Variables

We controlled for several variables that may affect the proposed relationships but that were not of direct theoretical interest. We controlled for respondents’ age and gender because both variables have been found to affect burnout in physicians and nurses (e.g., Maslach and Leiter, 2017; West et al., 2018). We also controlled for the participants’ years of professional experience because their predictive role in burnout in these professionals has been demonstrated previously (e.g., Marôco et al., 2016; Meira et al., 2017).

Income was also included because it is an important predictor of well-being (Lucas and Schimmack, 2009).

We controlled for religion and political orientation of the participants because religion is an important predictor of well-being (Koenig, 2012) and is usually associated with a more right wing political orientation (Correia et al., 2018).

Finally, questions related to COVID-19 pandemic (trust in policies, tasks changed and isolation the family) that might have been additional stressors for the health care professionals were also included as control variables.

In sum, in the present study we expect workload to be a risk factor for both dimensions of burnout both in physicians and nurses; we expect justice perceptions and professional identification to be protectors of burnout. We do not make specific predictions for meaningful work and empathy, because previous studies have found they may be either protector or risk factors. Furthermore, we will test if all the previous associations continue to be significant over and above the control variables.

## Materials and Methods

### Participants

Table 1 presents the descriptive statistics for control variables and Table 2 presents the descriptive statistics for the theoretical variables.

**TABLE 1:** Descriptive statistics for control variables.

**Control variables**	**Physicians *N* = 229**	**Nurses *N* = 268**
**Sex (%)**		
Male	48	27
Female	52	73
Age (M/SD)	36.54 (10.72)	34.96 (9.52)
**Nationality (%)**		
Portuguese	94.8	99.3
Other	5.2	0.7
Religiosity (M/SD)	2.84 (1.17)	2.84 (1.07)
Political orientation (M/SD)	3.08 (0.87)	3.00 (0.79)
years of professional experience (M/SD)	11.23 (10.65)	11.72 (9.24)
**Sector (%)**		
Public	58.5	58.2
Private	7.9	21.6
Both	33.6	20.1
Income (M/SD)	3.11 (0.71)	2.73 (0.70)
Trust in policies (COVID-19) (M/SD)	4.50 (0.72)	4.44 (0.72)

**TABLE 2:** Descriptive statistics for theoretical variables.

**Theoretical variables**	**Physicians *N* = 229**	**Nurses *N* = 268**
Exhaustion (M/SD)	3.07 (0.65)	3.10 (0.60)
Disengagement (M/SD)	2.69 (0.71)	2.76 (0.70)
Workload (M/SD)	3.06 (1.01)	3.32 (1.00)
Empathy cognitive (M/SD)	4.05 (0.42)	3.95 (0.36)
Affective empathy (M/SD)	2.98 (0.77)	2.94 (0.79)
Meaningful work (M/SD)	4.58 (0.55)	4.47 (0.55)
Justice of colleagues (M/SD)	3.92 (0.65)	3.84 (0.57)
Patient justice (M/SD)	4.18 (0.64)	4.06 (0.66)
Family patient justice (M/SD)	4.02 (0.70)	3.85 (0.74)
Distributive justice (M/SD)	2.22 (0.87)	1.93 (0.74)
Procedural justice (M/SD)	2.90 (0.88)	2.93 (0.80)
Professional identification (M/SD)	4.65 (0.65)	4.50 (0.70)

The sample of the present study was composed by 229 physicians (aged between 23 and 70 years old, *M* = 36.54; *SD* = 10.72; 48% male and 52% female) and 268 nurses (aged between 22 and 69 years old, *M* = 34.96; *SD* = 9.52; 26.9% male and 73.1% female).

About 58.5% of physicians and 58.2% of nurses worked in the public sector, 7.9% physicians and 21.6% nurses worked in the private sector and 33.6% physicians and 20.1% nurses worked in both sectors.

The average number of years of participants exercising their profession was around 11 years (*M* = 11.23; *SD* = 10.65, for physicians; *M* = 11.72, *SD* = 9.24, for nurses). The participants were from all regions in the country, but mostly from Metropolitan Area of Lisbon (43.7% physicians; 46.3% nurses), Center (15.3% physicians; 19.4% nurses) and North (17.9% physicians; 13.4% nurses) (Table 3).

**TABLE 3:** Percentage distribution of participants by area of residence.

**Area of residence**	**Physicians (%) *N* = 229**	**Nurses (%) *N* = 268**
North	17.9	13.4
Center	15.3	19.4
South	11.4	11.2
Metropolitan area Lisbon	43.7	46.3
Metropolitan area Oporto	8.7	5.6
Azores/Madeira Island	1.3	1.1
Other countries	1.7	3.0

A total of 43.2% physicians and 39% nurses were isolated from their nuclear family due to the COVID-19 pandemic and about 82.5% physicians and 60.4% nurses have changed their functions due to this pandemic. Most participants considered that the policies adopted by their country to combat the COVID-19 pandemic were adequate (physicians: *M* = 4.50, *SD* = 0.72; nurses: *M* = 4.44, *SD* = 0.72).

### Procedure

This study received Ethical approval by the Portuguese Order of Psychologists (OPP—Ordem dos Psicólogos Portugueses), in the framework of an initiative to support scientific research in health psychology and behavior change (Via Verde de Apoio OPP para a Investigação Científica em Saúde Psicológica e Mudança Comportamental).

An online survey was created using Qualtrics and participants were recruited via Facebook and LinkedIn^1^. The link for the study was also available at the website of the Portuguese Order of Psychologists.

At the beginning of the survey, the participants were informed about the general purpose of the study. Participants were informed that the study was non-invasive, there were no physical, financial, social, legal or other risks connected with the study and the results would be analyzed anonymously. It was also explained that they could withdraw from the study by closing the web browser without their responses being recorded.

The contact of the person responsible for the project was given in case they wished to obtain additional information or had any questions about the study.

After providing informed consent and agreeing to participate, they were presented with the main measures. In the last block of the survey, participants were asked to provide demographic and professional information and questions related to the Pandemic COVID-19.

At the end, the participants were debriefed and the theoretical variables of the study were indicated. The participants were thanked for their participation, and the contact of the person responsible for the project was again provided.

The average completion time of the survey was 10 min. The data were collected during 29 days (between the 45th and the 74th days after the first diagnosed case of COVID-19 in Portugal, in most of this time the country was in lockdown).

### Measures

#### Burnout

We used the Portuguese adaption (e.g., Sinval et al., 2019), of the Oldenburg Burnout Inventory (OLBI, Bakker et al., 2004). The OLBI has sixteen items and consists of two dimensions with eight items each: exhaustion (e.g., “There are days when I feel tired before I arrive at work,” physicians α = 0.83; nurses α = 0.81) and disengagement (e.g., “It happens more and more often that I talk about my work in a negative way,” physicians α = 0.86; nurses α = 0.83). Responses were given on a five-point scale, 1 = “totally disagree” to 5 = “totally agree.”

#### Justice Perceptions

Distributive justice was assessed with three items (e.g., “In general, the rewards I receive are fair,” physicians α = 0.82; nurses α = 0.82) taken from the five item distributive justice scale developed and validated by Rego to the Portuguese population (2000). The items were answered in a scale from 1 = “totally disagree” to 5 = “totally agree.”

Procedural justice was assessed with three items (e.g., “My organization has a mechanism that allows employees to appeal decisions”) taken from the four item scale of procedural justice scale of Rego (2000) and with three items (e.g., “My superior treats me with respect and consideration”) taken from the five item scale of interactional justice developed and validated by Rego (2000). An aggregated index of procedural justice was composed with the mean of the items used to measure procedural justice and interactional justice (physicians α = 0.88; nurses α = 0.88). We thus avoided multicollinearity issues, due to the high correlation between procedural and interactional justice (physicians *r* = 0.68; nurses *r* = 0.66).

Justice of colleagues was measured using two items (e.g., “My colleagues appreciate my work,” “My colleagues acknowledge my work,” physicians α = 0.82; nurses α = 0.84).

Patients justice was measured using two items (“My patients recognize my work,” “My patients acknowledge my work”). Justice of patient family members was assessed using two items “My patients’ relatives recognize my work,” “My patients’ relatives acknowledge my work”). For these items, a five-point response scale was used: 1 = “totally disagree” to 5 = “totally agree.” Because there was a very high correlation between patient justice and family patients justice (Table 4, physicians *r* = 0.75; Table 5, nurses *r* = 0.76), and to avoid multicollinearity issues, we calculated an aggregated index of patient and family patient’s justice (physicians α = 0.92; nurses α = 0.91) to use on the regression analysis.

**TABLE 4:** Correlations between study variables in physicians (*N* = 229).

	**1**	**2**	**3**	**4**	**5**	**6**	**7**	**8**	**9**	**10**	**11**	**12**	**13**	**14**	**15**	**16**	**17**	**18**	**19**	**20**
1. Exhaustion	–																			
2. Disengagement	0.58***	–																		
3. Sex	0.10	−0.14*	–																	
4. Age	−0.14*	–0.10	–0.10	–																
5. years of professional experience	–0.10	–0.08	–0.08	0.97***	–															
6. Income	−0.24***	−0.17**	–0.02	0.09	0.11	–														
7. Religiosity	–0.06	–0.13	0.17*	0.17*	0.18**	–0.03	–													
8. Political orientation	–0.04	0.06	–0.00	–0.05	–0.02	–0.07	0.20**	–												
9. Isolated the family (COVID-19)	–0.06	–0.00	–0.01	0.24***	0.24***	0.10	0.23**	–0.03	–											
10. Task changes (COVID-19)	–0.03	–0.04	–0.09	0.11	0.10	0.06	–0.06	–0.07	0.01	–										
11. Trust in policies (COVID-19)	−0.16*	−0.14*	–0.06	0.05	0.02	0.08	0.01	−0.16*	0.10	–0.10	–									
12. Workload	0.39***	0.25***	0.05	−0.17*	−0.15*	−0.19**	–0.11	0.08	–0.07	–0.02	–0.05	–								
13. Empathy cognitive	–0.03	−0.14*	0.17**	–0.05	–0.07	0.01	–0.01	–0.05	−0.18**	0.04	0.05	0.01	–							
14. Empathy affective	0.06	–0.00	0.00	–0.01	–0.02	0.10	–0.02	–0.12	–0.04	–0.01	0.10	0.00	0.01	–						
15. Meaningful work	–0.07	−0.26***	0.15*	0.00	0.01	0.07	0.12	0.11	–0.06	–0.01	0.07	–0.06	0.23**	0.03	–					
16. Justice of colleagues	−0.14*	−0.21**	–0.11	–0.07	–0.09	0.16*	–0.01	–0.00	–0.03	–0.13	0.08	–0.03	0.12	0.25***	0.26***	–				
17. Patient justice	–0.03	−0.13*	–0.03	0.12	0.10	0.05	0.00	0.07	–0.10	0.04	–0.09	0.01	0.24***	0.14*	0.24***	0.25***	–			
18. Family patient justice	–0.11	−0.21**	–0.06	0.15*	0.13	0.10	0.03	–0.05	–0.07	0.04	–0.05	–0.07	0.16*	0.15*	0.08	0.24***	0.75***	–		
19. Distributive justice	−0.33***	−0.22**	–0.03	0.10	0.10	0.37**	0.04	–0.08	0.19**	0.02	0.17*	−0.35***	0.01	0.19**	–0.08	0.09	0.01	0.08	–	
20. Procedural justice	−0.44***	−0.47***	–0.05	–0.02	–0.02	0.18**	0.17*	0.01	0.08	–0.01	0.16*	−0.35***	0.10	0.13	0.15*	0.29***	0.01	0.09	0.43***	–
21. Professional identification	−0.26***	−0.41***	0.08	0.12	0.10	0.09	0.19**	0.11	–0.06	–0.05	0.00	–0.02	0.11	–0.02	0.31***	0.16*	0.25***	0.17**	0.07	0.12

For all measures, scores were computed by averaging across items, with higher scores indicating stronger endorsement of the construct. For sex, 1 indicates “male” and 2 “female.” *p < 0.05; **p < 0.01, ***p < 0.001.

**TABLE 5:** Correlations between study variables in nurses (*N* = 268).

	**1**	**2**	**3**	**4**	**5**	**6**	**7**	**8**	**9**	**10**	**11**	**12**	**13**	**14**	**15**	**16**	**17**	**18**	**19**	**20**
1. Exhaustion	–																			
2. Disengagement	0.49***	–																		
3. Sex	0.16**	–0.01	–																	
4. Age	−0.17**	–0.09	−0.16*	–																
5. years of professional experience	−0.19**	–0.09	–0.11	0.93***	–															
6. Income	−0.29***	−0.21**	–0.03	–0.04	0.01	–														
7. Religiosity	–0.07	−0.19**	–0.02	0.18**	0.17**	–0.06	–													
8. Political orientation	0.00	0.02	0.00	0.08	0.08	0.00	0.14*	–												
9. Isolated the family (COVID-19)	–0.09	–0.06	–0.00	0.20**	0.18**	–0.07	0.14*	–0.02	–											
10. Task changes (COVID-19)	−0.13*	–0.10	–0.06	0.08	0.11	0.12	–0.04	–0.04	0.10	–										
11. Trust in policies (COVID-19)	−0.24***	−0.17**	0.02	–0.04	0.02	0.04	0.08	–0.12	0.02	0.07	–									
12. Workload	0.52***	0.40***	0.10	−0.16**	−0.15*	−0.21**	−0.13*	–0.07	–0.11	−0.15*	−0.20**	–								
13. Empathy cognitive	0.11	–0.02	0.08	0.02	0.05	−0.14*	0.10	0.04	–0.07	−0.23***	0.08	0.10	–							
14. Empathy affective	0.23***	0.08	0.15*	–0.05	–0.02	–0.05	–0.02	–0.03	0.05	–0.04	–0.01	0.11	0.06	–						
15. Meaningful work	−0.16*	−0.36***	0.04	0.06	0.08	0.04	0.12*	−0.16**	0.07	0.01	0.01	–0.12	0.22***	0.00	–					
16. Justice of colleagues	−0.15*	−0.32***	–0.07	0.13*	0.09	–0.01	0.12	0.11	–0.04	0.04	0.04	−0.14*	0.09	–0.07	0.20**	–				
17. Patient justice	−0.13*	−0.27***	–0.11	0.06	0.06	–0.08	0.16**	–0.02	–0.04	–0.04	0.16**	–0.12	0.12*	–0.08	0.18**	0.21**	–			
18. Family patient justice	−0.15*	−0.26***	–0.05	0.01	0.04	–0.05	0.14*	0.08	–0.01	0.07	0.16**	−0.15*	0.15*	–0.06	0.14*	0.26***	0.76***	–		
19. Distributive justice	−0.37***	−0.38***	–0.02	0.06	0.05	0.27***	0.10	0.05	0.07	0.05	0.16*	−0.48***	−0.20**	–0.10	0.01	0.03	0.05	0.08	–	
20. Procedural justice	−0.41***	−0.49***	−0.13*	–0.06	–0.04	0.21***	0.05	0.07	0.04	0.05	0.19**	−0.42***	−0.12*	–0.10	0.13*	0.20**	0.10	0.14*	0.45***	–
21. Professional identification	−0.13*	−0.45***	0.02	0.06	0.01	0.01	0.04	–0.10	–0.01	–0.02	0.05	–0.09	0.08	0.02	0.32***	0.27***	0.15*	0.13*	0.13*	0.14*

For all measures, scores were computed by averaging across items, with higher scores indicating stronger endorsement of the construct. For sex, 1 indicates “male” and 2 “female.” *p < 0.05; **p < 0.01, ***p < 0.001.

#### Professional Identification

This construct was measured with one-item measure (“I identify with my profession”) and the responses were given on a five-point scale ranging from 1 (“totally disagree”) to 5 (“totally agree”). This item was adapted from the one item measure of organizational identification used by Postmes et al. (2012) (“I identify with the organization I work for”).

#### Meaningful Work

Meaningful Work was evaluated with two items (“The work I do serves a greater purpose,” “I know my work makes a positive difference in the world”; physicians α = 0.77; nurses α = 0.72) taken from The Work and Meaning Inventory (WAMI) (Steger et al., 2012): with a five-point response scale ranging from 1 = “totally disagree” to 5 = “totally agree.”

#### Empathy

Empathy was measured using the Portuguese Adaptation of the Basic Empathy Scale short version (BES-A) (Pechorro et al., 2018). This version is a translation and validation of a shorter version (Salas-Wright et al., 2012) of the Basic Empathy Scale (BES) (Jolliffe and Farrington, 2006). This BES-A version has seven items, with three items for the affective dimension (e.g., “After being with a friend who is sad about something, I usually feel sad,” physicians α = 0.78; nurses α = 0.80) and four items for the cognitive dimension (e.g., “I can often understand how people are feeling even before they tell me,” physicians α = 0.81; nurses α = 0.69). The items were answered on a five-point response scale: 1 = “totally disagree” to 5 = “totally agree.”

#### Workload

Workload was evaluated with one item taken from The Areas of Worklife Scale (AWS) (“I have enough time to do what’s important in my job”—recoded) (Leiter and Maslach, 2003), on a five-point response scale: 1 = “totally disagree” to 5 = “totally agree.”

#### Control Variables

Income was measured with an item adapted from the European Social Survey (2018): “Which of the following descriptions is closest to your current income?” with a four statements response scale, 1 = “It is very difficult to live with my current income”; 2 = “It is difficult to live on my current income”; 3 = “My current income is enough to live”; 4 = “My current income allows me to live comfortably.”

Religiosity was measured with an item adapted from the European Social Survey (2018): “Regardless of whether you belong to a particular religion, how religious would you say you are?” with a five-points answer scale ranging from 1 “not religious at all” to 5 “very religious.”

Political Orientation was measured with an item adapted from the European Social Survey (2018). (“In politics people sometimes talk of ‘left’ and ‘right.’ Where would you place yourself on this scale, where 1 means the left and 5 means the right?”).

Three items refering to specific factors related to COVID-19 Pandemic were used. Trust in policies to combat the COVID-19: “To what extent do you consider that the measures to deal with this pandemic in your country are adequate?” from 1 “nothing” to 5 “very much”; task changes: “Has the COVID-19 pandemic changed your functions?” Yes/No answer; and isolation from family: “Are you isolated from your nuclear family due to COVID-19?” Yes/No answer).

## Results

### Preliminary Analysis

The descriptive statistics for all variables are given in Tables 1, 2. Physicians (*M* = 3.07, *SD* = 0.65) and nurses (*M* = 3.10, *SD* = 0.60) had significantly higher levels of exhaustion than disengagement (*M* = 2.69, *SD* = 0.71, for physicians; *M* = 2.76, *SD* = 0.70, for nurses), [physicians *t*(228) = 9.28, *p* < 0.001; nurses *t*(267) = 8.41, *p* < 0.001].

First, we examined the pattern of correlations between the variables under study separately for physicians and nurses. Table 4 (physicians) and Table 5 (nurses) depict correlations between study variables.

For physicians, significant negative correlation for exhaustion and disengagement were found with the following variables: income, trust in policies (COVID-19), justice of colleagues, distributive justice, procedural justice, and professional identification. A positive significant association between workload and exhaustion and disengagement was obtained. Cognitive empathy, meaningful work, patient justice, and family patient justice were significantly and negatively correlated with disengagement. Being a man was a risk factor for disengagement.

For nurses, we found a negative significant correlations for both exhaustion and disengagement with income, trust in policies (COVID-19), meaningful work, justice of colleagues, patient justice, family patient justice, distributive justice, procedural justice, and professional identification. Furthermore, exhaustion was positively associated with affective empathy, and negatively associated with age, years of professional experience and task changes (COVID-19). A significant negative association between disengagement and religion was obtained. Being a woman was a risk factor for exhaustion. A positive association was also found between both exhaustion and disengagement with workload.

### Main Analysis

To clarify the relationships between our variables, multiple regression analyses were performed for each burnout dimension (exhaustion and disengagement) regarding each professional group. The variables were ordered in two blocks: control variables—Step 1 = sex, years of professional experience^2^, income, religiosity, political orientation, isolated from family, task changes, trust in policies to deal with COVID-19; and theoretical predictors—Step 2 = workload, cognitive empathy, affective empathy, meaningful work, justice of colleagues, patient and family patient’s justice, distributive justice, procedural justice, professional identification.

Therefore, we will study the explanatory power of theoretical predictors of exhaustion and disengagement in addition to that of control variables. For both subsamples, the results of Model 2 is presented in Table 6.

**TABLE 6:** Regressions: predicting burnout in physicians (*N* = 229) and nurses (*N* = 268).

	**Exhaustion**						**Disengagement**					
	
	**Physicians**			**Nurses**			**Physicians**			**Nurses**		
	
	**B**	**SEB**	**β**	**B**	**SEB**	**β**	**B**	**SEB**	**β**	**B**	**SEB**	**β**
**Step 1—Controlling variables**												
Sex	0.06	0.08	0.04	0.09	0.07	0.06	–0.22	0.08	−0.15**	–0.13	0.07	–0.08
years of professional experience	–0.00	0.00	–0.03	–0.01	0.00	−0.12*	–0.00	0.00	–0.06	–0.00	0.00	–0.03
Income	–0.08	0.06	–0.09	–0.14	0.05	−0.16**	–0.04	0.06	–0.04	–0.11	0.05	−0.10*
Religiosity	0.04	0.03	0.07	0.02	0.03	0.03	0.01	0.04	0.02	–0.06	0.03	−0.10*
Political orientation	–0.06	0.04	–0.07	0.01	0.04	0.02	0.07	0.05	0.09	0.03	0.04	0.03
Isolated from family (COVID-19)	0.00	0.08	0.00	–0.05	0.06	–0.04	0.04	0.08	0.03	–0.02	0.07	–0.01
Task changes (COVID-19)	–0.05	0.10	–0.03	–0.01	0.06	–0.01	–0.13	0.10	–0.07	–0.08	0.07	–0.05
Trust in policies (COVID-19)	–0.09	0.05	–0.10	–0.11	0.04	−0.13*	–0.08	0.06	–0.08	–0.02	0.05	–0.02
**Step 2—Theoretical predictors**												
Workload	0.15	0.04	0.24***	0.19	0.04	0.31***	0.06	0.04	0.09	0.08	0.04	0.11***
Cognitive empathy	0.02	0.09	0.01	0.08	0.09	0.05	0.02	0.10	0.01	–0.03	0.10	–0.01
Affective empathy	0.10	0.05	0.12*	0.11	0.04	0.15**	0.08	0.05	0.08	0.02	0.04	0.02
Meaningful work	0.08	0.07	0.07	–0.09	0.06	–0.08	–0.08	0.08	–0.06	–0.19	0.06	−0.15***
Justice of colleagues	–0.02	0.06	–0.02	–0.02	0.06	–0.02	–0.05	0.07	–0.05	–0.12	0.06	−0.10*
Patient and family patient’s justice	−01	0.06	–0.01	–0.03	0.05	–0.04	–0.09	0.07	–0.08	–0.13	0.05	−0.12***
Distributive justice	–0.05	0.05	–0.07	–0.05	0.05	–0.06	0.01	0.05	0.02	–0.11	0.05	−0.12*
Procedural justice	–0.22	0.05	−0.30***	–0.11	0.05	−0.15*	–0.32	0.05	−0.39***	–0.23	0.05	−0.27***
Professional identification	–0.22	0.06	−0.22***	–0.03	0.05	–0.03	–0.35	0.06	−0.31***	–0.29	0.05	−0.28***
Constant	4.81	0.73		3.96	0.66		6.76	0.77		7.67	0.70	
R^2^	0.36			0.42			0.42			0.52		
R^2^ adjusted	0.31			0.38			0.37			0.49		
R^2^ change	0.26***			0.22***			0.33***			0.40***		

B, Unstandardized coefficients; β, Unstandardized coefficients. R^2^ corresponds to Step 2; R^2^ change between Step 1 and Step 2. For all measures, scores were computed by averaging across items, with higher scores indicating stronger endorsement of the construct. For sex, 1 indicates “male” and 2 “female.” *p < 0.05; **p < 0.01; ***p < 0.001.

In physicians, 36% of the variance in exhaustion was predicted by workload (beta = 0.24; *p* < 0.001), affective empathy (beta = 0.12; *p* < 0.05), procedural justice (beta = −0.30; *p* < 0.001) and by professional identification (beta = −0.22; *p* < 0.001). Higher workload and affective empathy significantly predicted higher exhaustion. Higher procedural justice and professional identification significantly predicted lower exhaustion.

For nurses, 42% percent of the variance in exhaustion was predicted by years of professional experience (beta = −0.12; *p* < 0.05), income (beta = −0.16; *p* < 0.01), trust in policies (COVID-19) (beta = −0.13; *p* < 0.05), workload (beta = 0.31; *p* < 0.001), affective empathy (beta = 0.15; *p* < 0.01) and procedural justice (beta = −0.15; *p* < 0.05). Higher workload and affective empathy significantly predicted higher exhaustion. Age, higher income, higher trust in policies (COVID-19) and higher procedural justice significantly predicted lower exhaustion.

For physicians, 42% of the variance in disengagement was predicted by sex (beta = −0.15; *p* < 0.01), procedural justice (beta = −0.39; *p* < 0.001) and professional identification (beta = −0.31; *p* < 0.001). Being a male, a perception of higher procedural justice and professional identification significantly predicted lower disengagement.

Finally, 52% of the variance in disengagement in nurses was predicted by income (beta = −0.10; *p* < 0.05), religiosity (beta = −0.10; *p* < 0.05), workload (beta = 0.11; *p* < 0.05), meaningful work (beta = −15; *p* < 0.01), justice of colleagues (beta = −0.10; *p* < 0.05), patient and family patient’s justice (beta = −0.12, *p* < 0.05), distributive justice (beta = −12; *p* < 0.05), procedural justice (beta = -0.27; *p* < 0.001) and professional identification (beta = −0.28; *p* < 0.001). High income, religiosity, meaningful work, justice of colleagues, patient and family patient’s justice, distributive justice, procedural justice, and professional identification significantly predicted lower disengagement. Higher workload significantly predicted higher disengagement.

Given the results consistently found the unique impact of procedural justice and professional identification as burnout protectors (except for exhaustion in nurses), and workload as a core risk factor (except for disengagement in physicians), we tested three possible models of the relation between these variables. We used Hayes’s (2013) Multiple Mediation macro (5,000 iterations; bias corrected) and we included the significant predictors found in the regression analysis (Table 6) as covariates.

Based on the group-value model, we first tested the possible indirect effect of procedural justice on exhaustion through professional identification in physicians and disengagement (both for physicians and nurses). None of the indirect effects of procedural justice on burnout through professional identification was significant (exhaustion in physicians: beta = −0.02, CI _95__%_ [−0.06, 00]; disengagement in physicians beta = −0.04, CI _95__%_ [−0.09, 0.00]; disengagement in nurses, beta = −0.01, CI _95__%_ [−0.05, 0.03].

We also tested a model where professional identification has an indirect effect on burnout through workload (Avanzi et al., 2018). None of the indirect effects of professional identification on burnout through workload were significant (exhaustion in physicians: beta = −0.01, CI _95__%_ [−0.03, 04]; exhaustion in nurses: beta = −0.03, CI _95__%_ [−0.09, 02]; disengagement in physicians beta = 0.00, CI _95__%_ [−0.01, 0.03]; disengagement in nurses, beta = 0.00, CI _95__%_ [−0.01, 0.02].

Finally, we tested the possibility of procedural justice buffering the impact of workload on burnout. The buffer effect of procedural justice on the relation between workload and burnout was only obtained for disengagement in nurses (beta = −0.11, CI _95__%_ [−0.18, −0.03]. In this sample, when procedural justice was lower, higher workload is significantly associated with higher disengagement (beta = 0.16, CI _95__%_ [0.07, 0.26]), but when procedural justice was higher, workload did not affect disengagement (beta = −0.01, CI _95__%_ [−0.10, 0.08]).

For physicians we did not obtain support for the buffer effect of procedural justice on workload, neither for exhaustion (beta = −0.02, CI _95__%_ [−0.10, 0.05]), nor for disengagement (beta = −0.09, CI _95__%_ [−0.18, 0.00]).

## Discussion

This paper aimed to test and compare the unique and predictive impact of workload, empathy, meaningful work, perceptions of justice, and professional identification on burnout (exhaustion and disengagement) of physicians and nurses in times of COVID-19 pandemic. These variables have been shown to be important predictors of burnout, but have not been considered together in a same study. Furthermore, we also controlled for individual variables, that could impact on that relations: demographic, ideological, and related with COVID-19 pandemic.

The results of correlations generally confirmed the ones of previous research. However, the testing and comparison of the unique impact of each of these variables revealed that the predictors considered are not equally important. We obtained a pattern of results that shows some predictors are common to both dimensions of burnout both in physicians and nurses, and a specificity of some of the predictors for each of the dimensions of burnout and that differ between physicians and nurses.

Workload was positively associated with both dimensions of burnout both in physicians and nurses (e.g., West et al., 2018; Dubale et al., 2019), and even when all variables were considered, workload remained a significant risk factor except for disengagement in physicians.

Justice perceptions were found to be differently associated with burnout, all of them as protective factors when significant. Procedural justice, distributive justice and justice of colleagues were negatively associated with both dimensions of burnout both in physicians and nurses. Justice of patients and family patient justice were negatively associated with exhaustion (nurses) and disengagement (physicians and nurses). These results confirm the research previously conducted by Moliner et al. (2005) demonstrating the protective role of justice in burnout. Furthermore, procedural justice was a unique and significant protector for the two dimensions of burnout in physicians and nurses. This is in line with the research that shows the importance of procedural justice for good functioning in organizations and well-being (e.g., Elovainio et al., 2002a).

Professional identification was correlated negatively with both dimensions of burnout both in physicians and nurses. These results support the hypothesis of professional identification as protective factor of burnout, as demonstrated in the study by Avanzi et al. (2018), and it remained a significant and unique protective factor for distancing (both in physicians and nurses) and for exhaustion in physicians, but not for exhaustion in nurses, confirming the important role of social identity for the protection against burnout.

For the disengagement of nurses, more dimensions of justice besides the procedural one remained significant protective factors when all variables were considered, namely distributive justice, justice of colleagues, and patient and family patient’s justice.

Meaningful work was negatively associated with exhaustion (nurses) and disengagement (physicians and nurses). These results support our hypothesis and confirm previous research on the premise of meaningful work as protective factor for burnout (e.g., Borritz et al., 2005) in physicians (e.g., Rasmussen et al., 2015) and nurses (e.g., Tei et al., 2014). However meaningful work only remained a significant predictor of disengagement in nurses.

For empathy the results support the hypothesis of empathy as risk factor, specifically affective empathy for exhaustion in nurses, and cognitive empathy as protective factor for disengagement in physicians. When all the variables were considered, affective empathy emerged as risk factor for exhaustion, both in physicians and nurses.

Income was a risk factor for both dimensions of burnout in nurses, which might be explained by the lower incomes of nurses in Portugal and by the fact that, for people with lower incomes, income is more strongly related with well-being than for people with higher incomes (Lucas and Schimmack, 2009). Indeed, income is a resource that helps individuals to overcome inconveniences and hassles, that allow individuals to obtain paid help for less enjoyable activities, such as chores, and also to engage in enjoyable activities (for a review, see Tay et al., 2017). Future studies should try to further investigate these associations.

Given the results consistently found the unique impact of procedural justice and professional identification as burnout protectors (except for exhaustion in nurses), and workload as a core risk factor (except for disengagement in physicians) we tested three possible models of the relation between these variables.

The first analysis was based on the group-value model. We tested the possible indirect effect of procedural justice on burnout through professional identification. However, our data did not support for the application of the group-value model to predict burnout in the present sample of physicians and nurses.

The second analysis was based on the model where professional identification has an indirect effect on burnout through workload (Avanzi et al., 2018). Again, our data did not support for the this model.

The third analysis, tested the possibility that procedural justice could act as a buffer and be a moderator of the impact of workload on burnout. Only for disengagement in nurses it was found that high procedural justice may decrease the impact of workload on burnout.

This study has important implications for interventions because it suggests several focus that might be simultaneously and independently addressed: decreasing workload and promoting procedural justice and professional identification, seem to be central to interventions for reducing the risk of burnout or preventing it from occurring in the first place. The fact that these factors are related with organizational issues goes in the same line that burnout intervention may be done at the level of the working conditions (Maslach and Leiter, 2015) and not mainly at the level of the treatment of the individual (Maslach et al., 2001) as it still happens (e.g., Hue and Lau, 2015; Castanheira, 2020).

This study has of course some limitations. Some of the constructs were assessed with few items, because we had many variables and the measurement of all of them with the entire scales would result in a very long questionnaire that would certainly discourage participants from answering. However, the measures of internal consistency of the shortened scales were very good.

A second limitation is related with the samples, that were convenience samples and the data were collected in a specific period of the pandemic. Nevertheless, the sample showed good variability in most of the sociodemographic, ideological and occupational variables, which supports its’ heterogeneity and therefore the possibility of generalizability of these results for similar samples.

One third limitation refers to the correlational design of this study, that limits the nature of the conclusions that can be drawn about the causal relations among variables. Nevertheless, considering psychosocial variables as predictors and burnout as an outcome is in line with previous research that tried to find the predictors of burnout (e.g., Demerouti et al., 2001).

A fourth limitation refers to the fact that all predictors and outcome variables were self-reported, which might lead to possible overestimation of the associations between them due to shared method variance.

Future studies should try to replicate these results with other samples of physicians and nurses and may also explore if the protective factors that we found in this study extend to other occupations in contexts and times less affected by the specificities of adapting to a Pandemic.

We strongly believe that this paper can contribute to encourage research to focus on the variables that are the stronger predictors of burnout and to stimulate the test of models that consider how these variables might relate. According to our results, the core variables seem to be procedural justice, professional identification and workload, and act independently of each other. With the continuation of the pandemic and the persistence of the increased stress on the health care systems, we recommend that procedural justice and professional identification should receive special attention of interventions in the Heath Care Services. However, we do not intend to devalue the importance of recommending the reduction of the workload to which these professionals are subject to. We think the study of protective factors is important, but they should not devalue the relevance and responsibility of organizations to reduce risk factors, namely workload.

## Data Availability Statement

The datasets presented in this article are not publicly available because the participants of this study did not agree for their data to be shared publicly. However, the datasets can be available under request directed to Isabel.correia@iscte-iul.pt.

## Ethics Statement

This study received Ethical approval by the Portuguese Order of Psychologists (OPP – Ordem dos Psicólogos Portugueses), in the framework of an initiative to support scientific research in health psychology and behavior change (Via Verde de Apoio OPP para a Investigac̨ão Científica em Saúde Psicológica e Mudanc̨a Comportamental). The participants provided their electronic informed consent to participate in this study.

## Author Contributions

IC planned the study and selected the variables and the instruments. IC and AA contributed to the writing of all parts of the manuscript. AA put up the questionnaire in the Qualtrics platform and recruited the participants via Facebook and LinkedIn. Both authors contributed to the article and approved the submitted version.

## Conflict of Interest

The authors declare that the research was conducted in the absence of any commercial or financial relationships that could be construed as a potential conflict of interest.

## Footnotes

1 We did not address specific groups of nurses and physicians. We addressed nurses and physicians individually and, in many cases, they shared the link with other professionals. Although the link of the questionnaire was available in the Portuguese Order of Psychologists, this happened only in the last days of the collection of data, and the specific location on that website is not specifically intended to be consulted by physicians and/or nurses.

2 We did not include age in the regression to avoid multicollinearity issues due to its’ high correlation with years of professional experience.
